# Editorial

**Published:** 1903-08-15

**Authors:** 


### EDITORIAL.

The Michigan State Dental Association met this year at Petoskey, a fine summer resort in the northern part of the State. There was some disappointment in the number of those who attended from the northern part of the State. There were but two dentists from the upper peninsula present, and both of these came from the eastern part of the peninsula. There was also a meager attendance from the upper part of the southern peninsula. Indeed, had it not been for the large numbers who came from the southern part of the State the meeting would have been a failure, so far as attendance was concerned. The program was excellent. The papers were all of an unusually high order, and were well discussed. The clinics were exceptionally good, and almost every one promised materialized. The next meeting will probably be held in Lansing. The officers elected were: Dr. C. C. Noble, President; Dr. F. H. Essig, Vice-President; Dr. J. J. Green, Secretary; Dr. J. W. House, Treasurer; Dr. H. K. Lathrop, Jr., Law Trustee. The New Arlington Hotel, where the meeting was held, was commodious and well kept, and the management did everything possible to make every one have a good time. The weather was fine, and there was no reason why any one should complain. There was a considerable number of exhibitors of supplies, and their displays were, under the new system of management, given abundant opportunities for displaying their goods without encroaching on the sessions of the meeting. The new method of conducting the affairs of the Association prevented the usual waste of time in considering business matters in the open sessions. The local dentists did handsomely in the way of entertainment, boat and railway rides, and a banquet gave plenty of opportunity for social intercourse. The only criticism we heard made on the meeting was the lack of attendance of the dentists from the nearby territory. Just what the reason for this may be we can't say, we are sure it can be no fault of the officers of the

Association, as most of them traveled long distances and at considerable expense to attend the meeting. Probably it is because the men are not in the habit of attending meetings; if so, some plan of forming this habit ought to be inaugurated as soon as possible. It is a curious fact that there is not a local dental society north of Saginaw. It might be a good schéme for the new officers to set about organizing local or district societies in various parts of the State, where none exist, with the hope that they may may become important feeders to the State Association. The proceedings will be begun in this issue of the REGISTER.

—✦—

Wisconsin has a new dental law which goes into effect in June. It contains no remarkable provisions. The noticeable features are that it requires annual registration and payment of a fee of one dollar therefor. It prescribes that graduates of such schools as its board may believe reputable, and which have a seven months' term and a course of four years, with entrance to third year of high school for admission, may be registered without examination. At the same time it prescribes that undergraduates in attendance at Wisconsin colleges may be registered, providing they agree to continue their studies until they do graduate. Just why such a proposition as this is allowed to become a part of a dental statute we confess we are unable to comprehend. It is probably the result of political pressure brought to bear by dental colleges or their students. There can be no good reason for such a provision. In defining who are practitioners this law says that any one who manufactures artificial teeth is practising dentistry. If this be true the dental laboratory will have to close its doors or get workmen who are legalized practitioners; literally it would also compel the manufacturer of teeth to become a legalized practitioner. We presume, however, that this was not the intention of the framers of the law.

It seems almost impossible to get a law through our legislative bodies that can only be interpreted to do the right thing. Some one ought to write a book on what not to put into dental laws.

